# Quantification of myocardial perfusion using CMR with a radial data acquisition: comparison with a dual-bolus method

**DOI:** 10.1186/1532-429X-12-45

**Published:** 2010-07-23

**Authors:** Tae Ho Kim, Nathan A Pack, Liyong Chen, Edward VR DiBella

**Affiliations:** 1 Utah Center for Advanced Imaging Research, Department of Radiology, 729 Arapeen Dr. University of Utah, Salt Lake City, Utah 84108, USA; 2 Current Address: Department of Radiation Oncology, Stanford University, Stanford, CA 94305, USA

## Abstract

**Background:**

Quantitative estimates of myocardial perfusion generally require accurate measurement of the arterial input function (AIF). The saturation of signal intensity in the blood that occurs with most doses of contrast agent makes obtaining an accurate AIF challenging. This work seeks to evaluate the performance of a method that uses a radial k-space perfusion sequence and multiple saturation recovery times (SRT) to quantify myocardial perfusion with cardiovascular magnetic resonance (CMR).

**Methods:**

Perfusion CMR was performed at 3 Tesla with a saturation recovery radial turboFLASH sequence with 72 rays. Fourteen subjects were given a low dose (0.004 mmol/kg) of dilute (1/5 concentration) contrast agent (Gd-BOPTA) and then a higher non-dilute dose of the same volume (0.02 mmol/kg). AIFs were calculated from the blood signal in three sub-images with differing effective saturation recovery times. The full and sub-images were reconstructed iteratively with a total variation constraint. The images from the full 72 ray data were processed to obtain six tissue enhancement curves in two slices of the left ventricle in each subject. A 2-compartment model was used to determine absolute flows

**Results:**

The proposed multi-SRT method resulted in AIFs that were similar to those obtained with the dual-bolus method. Myocardial blood flow (MBF) estimates from the dual-bolus and the multi-SRT methods were related by *MBF_multi-SRT _*= 0.85*MBF_dual-bolus _*+ 0.18 (r = 0.91).

**Conclusions:**

The multi-SRT method, which uses a radial k-space perfusion sequence, can be used to obtain an accurate AIF and thus quantify myocardial perfusion for doses of contrast agent that result in a relatively saturated AIF.

## Background

Myocardial perfusion cardiovascular magnetic resonance (CMR) is a useful modality for measuring perfusion to detect coronary artery disease and myocardial ischemia [1,2]. Quantitative estimates of perfusion require that the gadolinium concentration in the arterial input function (AIF) and the myocardial tissue curves be known. This is achieved by estimating the T_1 _values at each time frame of the blood and tissue. If the pre-contrast T_1 _value of blood and tissue is also known, these T_1 _values can be used to calculate the gadolinium concentrations over time. Equivalently, if the change in signal intensity does not saturate (meaning a loss in linearity between the gadolinium concentration and the change in the signal from pre-contrast), the change in signal intensity time curves can be used without explicit conversion to gadolinium concentration. However, the saturation of signal intensity in the blood during the first pass that occurs with most doses of gadolinium-based contrast agents hinders quantification. Dual-bolus methods with a 1 ml first injection [3] and with dilute matched volume injections [4-6] have been used to enable quantitative perfusion CMR. With these methods, an unsaturated AIF is obtained from a low dose injection, which is then scaled and used to replace the saturated AIF from the high dose injection. The requirement of two injections can be logistically challenging and may reflect different physiological states, particularly during vasodilation.

Recently, an alternative method for estimating T_1 _and thus gadolinium concentration from a single injection using a radial k-space acquisition was proposed [7]. That method created four sub-images each with different *effective *saturation recovery times (eSRTs) from 96 ray acquisitions to estimate the AIF accurately. Extracting images with different eSRTs from a single dataset is possible with a radial acquisition if one uses a subset of the rays to perform the image reconstruction, and it is assumed that each ray of the subset contributes equally to the image [7].

However, no measures of truth were used to evaluate the method *in vivo*. In this work, we employ a similar multiple effective saturation recovery time (multi-SRT) approach for dynamic estimation of contrast agent concentration in the left ventricle blood pool. The purpose of this study was to study the performance of the radial k-space perfusion sequence multi-SRT method by comparison to the established dual-bolus approach.

## Methods

### MR of Vials

To validate the multi-SRT method in a controlled setting, we estimated the T_1 _of vials with known concentrations of contrast agent using the saturation recovery radial acquisition described in the human protocol below. Eighteen vials with different concentrations of Gd-BOPTA (Multihance, Bracco Diagnostics Inc.) were prepared with the range of T_1 _spanning 10-1000 ms, covering the range of T_1 _of blood after a typical contrast injection. Each vial was positioned in a water bath containing gadolinium (~0.3 mM) to reduce magnetic susceptibility artifacts in the images. The image signal in a region of interest in each vial was averaged over 10 time frames to improve SNR. Reconstruction and multi-SRT processing were performed as detailed for the human studies below, with the T_1 _of the different vials being estimated jointly.

For comparison, T_1 _was also measured using an inversion recovery pulse sequence with three inversion times. The imaging parameters for this fast spin echo sequence were TR/TE = 4000/15 ms, TI = 25/100/300 ms, 9 echoes, and resolution 2.0 × 2.0 × 8.0 mm^3^.

### Human Subject CMR Protocol

Fourteen volunteers (8 female, 6 male, 52 ± 13 yrs, 183 ± 55 lbs) assumed to be without ischemia were imaged in this study. Informed consent from all the subjects was obtained in accordance with the University of Utah's human subject policies. All studies employed an extra "backcheck" or one-way valve on the saline side of the power injector, in addition to the standard backcheck valve on the contrast agent side of the power injector. This was to prevent any flow of contrast agent into the saline syringe, which has been observed to occur (though may be a negligible amount, depending on dose). All subjects were given a low dose (0.004 mmol/kg) of dilute (1/5 concentration) contrast agent (Gd-BOPTA, Multihance) and perfusion data acquired. Approximately 3-5 minutes later, a higher non-dilute dose (0.02 mmol/kg) was given and perfusion data again acquired. Both injections were given at 5 cc/sec and flushed with 25 ml of saline at 5 cc/sec. The studies were performed on a Siemens 3 Tesla Trio (n = 12) or a Siemens 3 Tesla Verio (n = 2) system (Siemens Medical Solutions, Erlangen, Germany), using the spine and body phased array coils. We employed a saturation recovery radial turboFLASH sequence that was very similar to the standard saturation recovery Cartesian turboFLASH sequences, just with a radial non-Cartesian readout. The radial readout acquired 72 rays over 180° in an interleaved manner (12 subsets of 6 rays each) approximately 10 msec after each saturation pulse to give the data for one slice. In order to sample more of k-space over time, a small offset of 180/(4*72) = 0.625° was used for sequential time frame acquisitions, with a period of four [7]. The acquisition parameters were TR/TE = 2.6/1.14 ms, prescribed flip angle = 14°, slice-thickness = 8 mm, number of readouts = 256, FOV = ~300 mm and ~70 time frames. Three to four slices were acquired per beat, and subjects were requested to breathe shallowly.

### Reconstruction of CMR Data

An iterative total variation constrained reconstruction was used with the 72 ray dynamic datasets to obtain the tissue enhancement curves. It was assumed for the dose used here that the tissue curves were not saturated. The reconstruction method was similar to that used in [8], although in this work a forward and inverse non-uniform FFT from [9] was used at every iteration. The same method was used to reconstruct three subsets of 24 rays each, in order to create three sub-images with different effective saturation recovery times. This is not possible with conventional Cartesian sequences - that is, using a subset of Cartesian lines (phase encodes) will result in aliased images that will mostly reflect edges if the center of k-space is not included. On the other hand, for radial acquisitions, any subset of rays can be used to reconstruct an image, albeit with more streaking than if all of the rays were used. The chosen rays will dictate the contrast of the sub-image because each ray samples the center of k-space which controls image contrast. Here, the three sub-images that were reconstructed were used solely to estimate the AIF.

### Analysis of CMR Perfusion Data

First, the 72 ray (full data) reconstruction was processed to obtain myocardial tissue curves and saturated AIFs. This was done with freely available custom software [10]. Manual correction of respiratory motion over the time frames was done with the software, as was selection of endocardial and epicardial borders in each short axis slice. A region of interest was also drawn on the left ventricular blood pool to provide the AIF signal. The mean tissue enhancement signals from six equiangular regions of the myocardium in each of the short axis slices were obtained from the full 72 ray images. Two relatively basal slices were selected for further processing. A uniform region of signal that comprised ~20% of the left ventricular blood pool and that did not contain papillary muscles was manually selected for the AIF.

### Multi-SRT Processing to obtain the AIF

The (saturated) AIFs from the three sub-images were created from the ROI chosen on the full data reconstruction. These three curves were then processed to obtain a single non-saturated AIF. Each of the sub-images has a different eSRT (43 ms for the first sub-image, 107 ms for the second sub-image, and 172 ms for the third sub-image), and can be expressed by the equation in [7] in terms of the readout flip angle *α*, a proton density term *M*, and T_1_. By simultaneously fitting the signal intensity from each sub-image curve to this signal equation, T_1 _was estimated for each time point. Only a single *α *and *M *were estimated for each time curve [7]. The T_1 _values at each time point were converted to concentration using the standard relation 1/T_1_-1/T_1_(0) = ß[Gd], with relaxivity ß = 5.5 mM^-1^s^-1 ^and T_1_(0) defined as the pre-contrast T_1 _value. This equation states that changes in (inverse) T_1 _from pre-contrast are linearly related to changes in gadolinium contrast agent concentration ([Gd]). T_1_(0) was obtained by fitting the low (1/5 concentration) dose blood pool multi-SRT curves. The average of the first 7 pre-contrast time points was used to find T_1_(0).

The multi-SRT AIF concentration curve was then scaled so that its average value in the final four time points matched the average of the four final frames of the blood signal from the full 72 ray image [7]. This procedure assumed that in the final frames there was no signal saturation of the blood signal in the full image. Note that this scaling step makes the choice of the relaxivity ß irrelevant in the conversion to concentration equation given above, and was done to match to the scale of the tissue curves.

### Dual-bolus Processing to obtain the AIF

For the dual-bolus method, the AIF for each subject was obtained from a manually drawn region of the left ventricular blood pool in the low dose 72 ray images. The AIF was upscaled by five to replace the volume matched high dose 72 ray AIF blood signal.

### Fitting for MBF

For perfusion quantification, the AIFs obtained from the multi-SRT method and the tissue enhancement curves from the 72 ray higher dose acquisition were input to a 2-compartment model [11] using the software mentioned previously [10] in order to obtain *K^trans^*, a measure of absolute MBF. The fitting procedure was repeated using the dual-bolus AIF.

### Statistical Analysis

Paired comparisons of MBF estimated using the two different AIFs were tested using the Students t-test. Pearson's correlation was calculated along with linear regression analysis, and Bland-Altman analysis was performed.

## Results

### Vials

**Figure 1:**
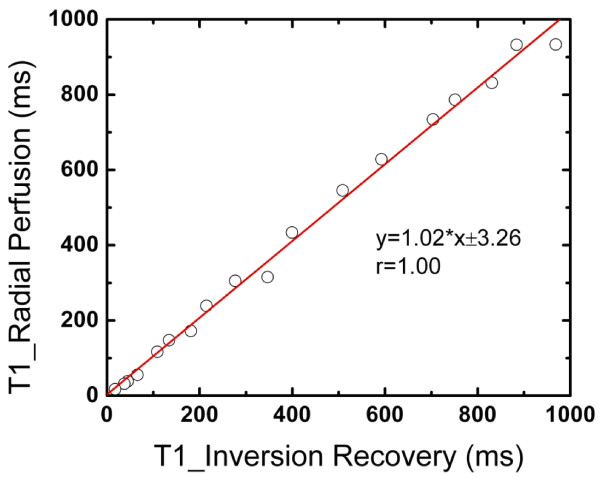
**The correlation between estimated T_1 _values in 18 vials using the multi-SRT method and the reference standard method (inversion recovery with three inversion recovery times)**.

T_1 _estimates from the 18 vials are presented in Figure 1. The multi-SRT method gave T_1 _values similar to the reference standard inversion recovery sequence, T_1*est *_= 1.02 T_1*ref*_+3.26 msec.

### Human Studies

**Figure 2:**
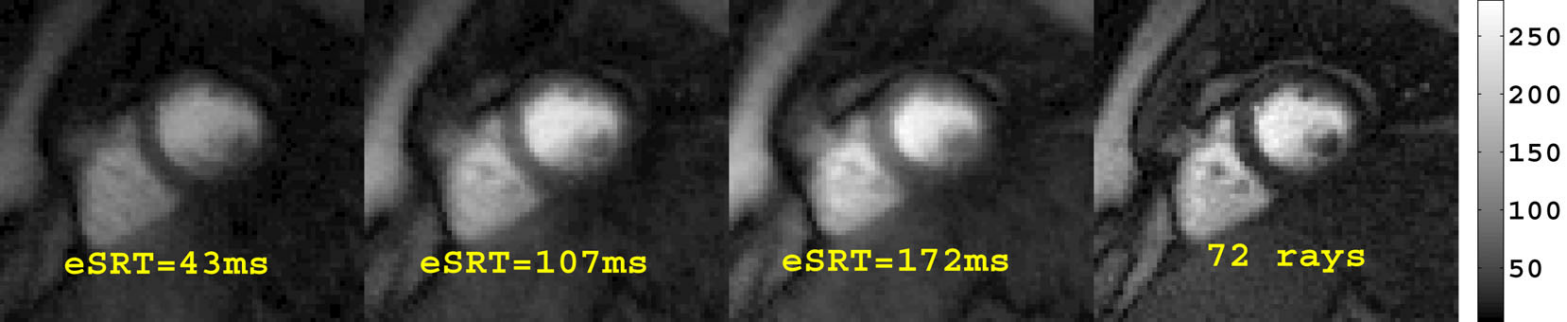
**Three sub-images with different eSRTs and the corresponding 72 ray combined image from a single time frame of a perfusion dataset are shown**.

**Figure 3:**
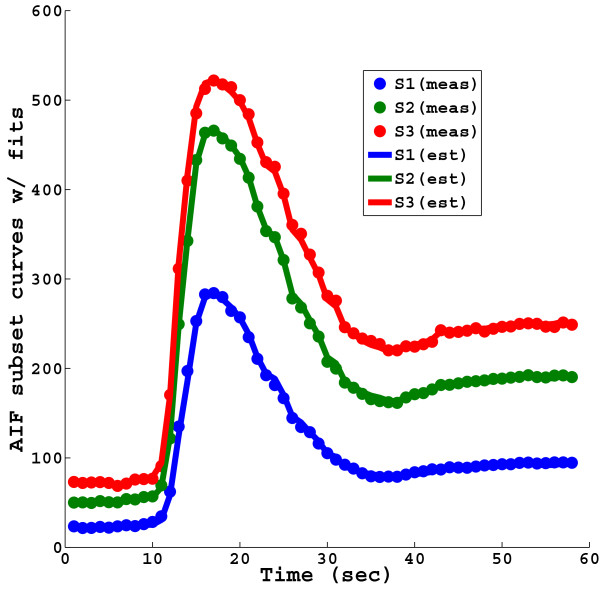
**The measured blood enhancement (saturated AIF) curves corresponding to the three sub-images with different eSRTs are shown, along with their fits obtained using the proposed multi-SRT measurement method**.

Three sub-images and the corresponding full 72 ray image from a single time frame of a typical perfusion dataset are presented in Figure 2. Although the 72 ray combined image shows better discrimination of the myocardium and the left ventricular blood pool, each of the sub-images is of sufficient quality to obtain the multi-SRT blood enhancement signals. An example of the time curves that are obtained from each of the sub-images is shown in Figure 3. The curve from the shortest eSRT has the lowest signal and the signal increases with eSRT. Fits to the curves from the different eSRT images are also shown.

**Figure 4:**
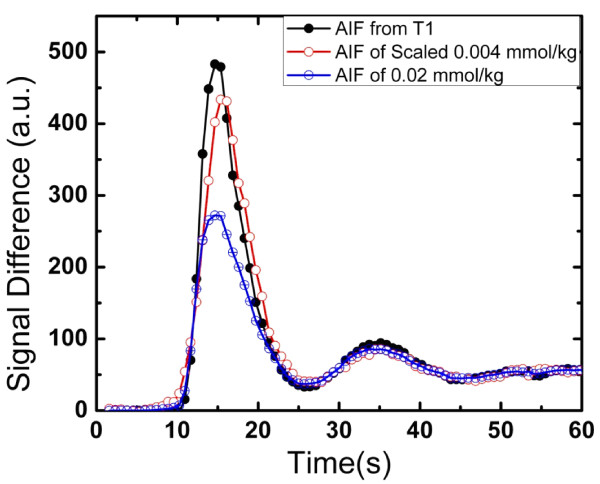
**A corrected AIF using the proposed multi-SRT method (black), along with the saturated AIF from the higher dose (0.02 mmol/kg) injection (blue) and the upscaled low dose AIF is shown (red)**.

**Figure 5:**
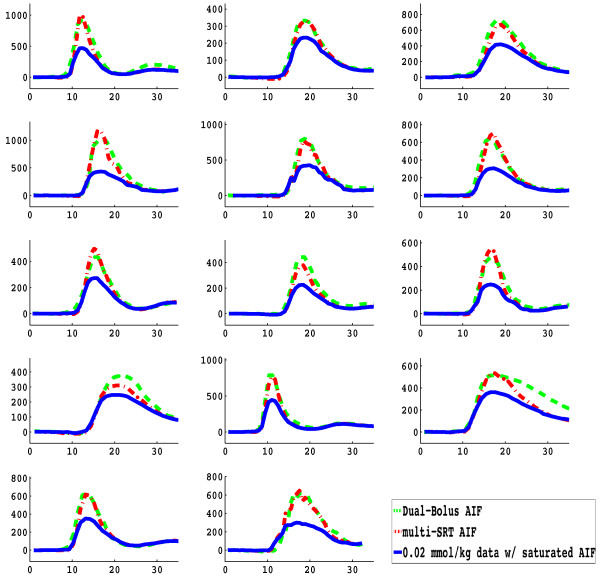
**Comparison of the corrected AIFs obtained using the multi-SRT method (red) and the low dose (0.004 mmol/kg) upscaled AIF used for the dual-bolus method (green), along with the saturated AIF from the high dose (0.02 mmol/kg) injection (blue), in all 14 subjects in the study**. Peak height of the saturated AIF varies from approximately 30% reduction of the dual-bolus or multi-SRT AIF, to 70% reductions.

In Figure 4, the corrected AIF using the proposed multi-SRT estimate method is shown, along with the saturated AIF from the "high" dose (0.02 mmol/kg) injection, and the upscaled low dose AIF from the dual-bolus method. The peak of the measured AIF from the 0.02 mmol/kg scan is approximately 40% below the upscaled low dose AIF. The proposed multi-SRT estimate method gave AIFs that were similar to those obtained with the dual-bolus method. The same type of comparison is shown for all 14 subjects in Figure 5. The other parameters estimated along with T_1 _and concentration were T_1_(0) = 2049 ± 434 ms, flip angle *α *= 8.5° ± 2.2°, and *M*= 812 ± 434.

**Figure 6:**
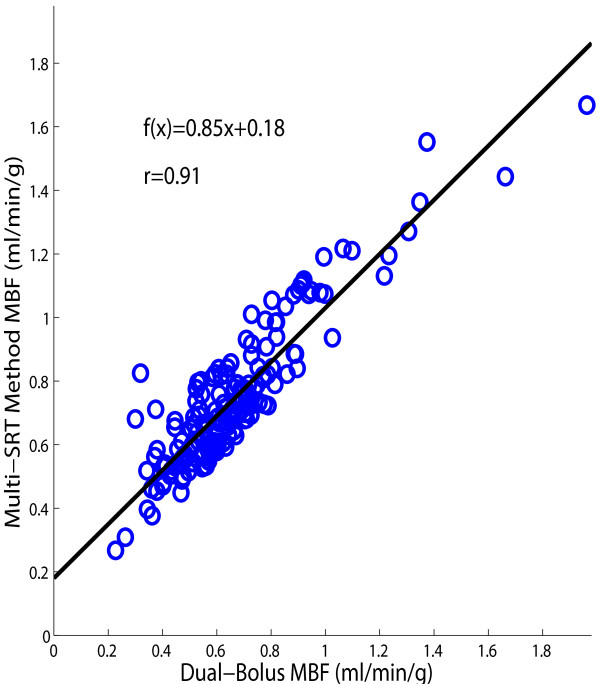
**The correlation between *MBF *estimates using the proposed multi-SRT measurement method and dual-bolus imaging**.

**Figure 7:**
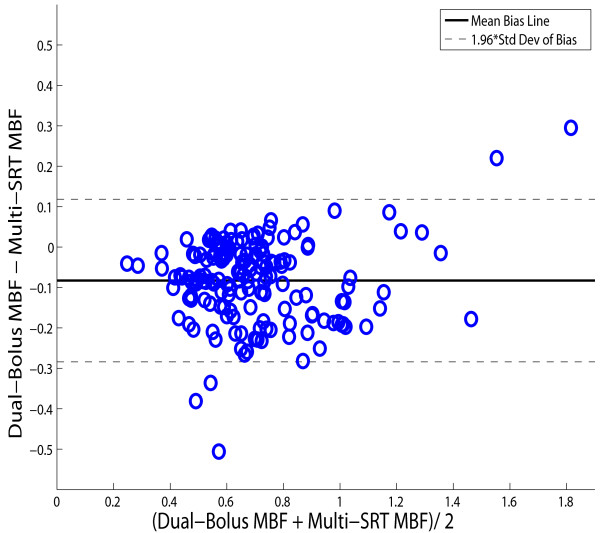
**A Bland-Altman plot showing the mean *MBF *estimates and the difference between *MBF *estimates from the multi-SRT method and dual-bolus imaging**.

Figure 6 shows the correlation between MBF values obtained using the multi-SRT method and the dual-bolus method. The MBF results from the proposed method were similar to the dual-bolus method: *MBF_multi-SRT _*= 0.85*MBF_dual-bolus _*+ 0.18 (r = 0.91). The aggregate MBF values estimated from the dual-bolus and the multi-SRT methods for all 14 subjects were 0.66 ± 0.24 and 0.75 ± 0.23 ml/min/g, respectively. These means were significantly different, *p *< 0.05. Figure 7 shows a Bland-Altman plot of the MBF estimates from the multi-SRT method and the dual-bolus method. For these 14 subjects, there was a mean overestimation in the multi-SRT MBF estimates of 0.08 ± 0.1 ml/min/g compared to the dual bolus results.

## Discussion

We report on a multi-SRT quantitative perfusion CMR method using a radial k-space acquisition with 72 rays. The method uses an iterative reconstruction with a total variation constraint, which has been shown to produce image quality similar or better than Cartesian acquisitions when only 24 rays per slice and multiple slices are acquired after a single saturation pulse and the first slice is not considered [8]. In this work, the reconstructed images used all 72 rays to obtain tissue enhancement curves, and used three subset images of 24 rays each to estimate the non-saturated AIF. There were relatively small signal changes from the second sub-image (eSRT = 107 ms) to the third sub-image (eSRT = 172 ms) suggesting that the third sub-image may not be essential for the approach. However, all three sub-images were used to estimate T_1 _in order to minimize the effects of undersampling and noise in the sub-images.

Other methods have been proposed to handle the saturated AIF problem. Low doses [12-17] can be used but give low signal. Analytical methods convert the measured signal intensities into gadolinium concentrations [18,19], but can be sensitive to noise when the signal-concentration relationship is not close to linear. Alternatively, two groups have developed custom sequences to measure an additional slice at low resolution and short SRT so that signal in that slice will be more linear with concentration and that slice can be used solely for the estimation of the AIF [20,21].

The proposed multi-SRT method was shown here to give accurate T_1 _estimates in vials, when compared to T_1 _values using an inversion recovery pulse sequence, (Figure 1). Ref. [7] found a similar relationship, although a 96 ray acquisition and four subsets were used, and a 3 D gradient echo acquisition was used as the reference standard.

When estimating gadolinium concentrations of the AIF in this work, we did not assume the prescribed flip angle was accurate, and instead estimated it as an unknown parameter in the multi-SRT curve-fitting process. Both spatial excitation (flip angle) variations and an imperfect slice profile likely played a role in the findings of a net flip angle for the blood region of 8.5 ± 2.2 degrees, instead of the 14° that was designated in the radial perfusion sequence. This nearly 40% reduction in flip angle is similar to that found previously in the heart at 3T [22]. A 23-48% variation in flip angle across the heart has also been reported at 3T [23]. Flip angle variation over the region of interest may have led to some variation in the multi-SRT gadolinium concentration estimates of the AIF. Changes in the AIF would alter the MBF estimates of all tissue curves of that subject.

T_1 _values prior to contrast injection were also estimated in this work. For reference, Lu et al. reported the T_1 _of ex-vivo bovine blood at 3T as 1664 ± 14 msec [24]. Studies in ex-vivo human blood at 37°C and 3T found 1932 ± 85 msec [25]. In our studies, the aggregate pre-contrast T_1 _estimates in the left ventricular blood pool were 2049 ± 434 ms, using the radial perfusion sequence. The mean of this result is in reasonable agreement; the relatively high standard deviation of the T_1 _estimates may be due to inter-subject variability.

As shown in Figs. 4-5, when no signal intensity correction is performed, the peak of the measured AIF from the 0.02 mmol/kg injection scan is saturated by approximately 30-70%. This is likely a larger effect than found with 0.02 mmol/kg scans using Gd-DTPA or Gd-DTPA-BMA (Magnevist or Omniscan), which have lower relaxivity [26]. Although done at 1.5T, others have reported that Gd-BOPTA saturates at significantly lower concentrations than Gd-DTPA [27].

The proposed multi-SRT method can correct for signal saturation in the AIF using a single injection of contrast agent that overcomes the complexity of multi-injection protocols. In addition, by using a single injection, there is no physiological change between the corrected AIF and the measured AIF that could be confounding with some dual-bolus imaging protocols. For example, in this study there were sometimes variations in a subject's heart rate (and likely the hemodynamics of blood and contrast agent flow in the left ventricle) during the two separate injections, indicating some degree of physiological change. With a single injection of contrast agent using the proposed method, these changes are not an issue. This is a larger concern in stress studies where it can be more difficult to have vasodilation kept constant, particularly if breath-holds also change perfusion to some degree.

The new multi-SRT method resulted in AIFs that were similar to those obtained with the dual-bolus method, and gave MBF estimates that on the average differed by less than 11%. This difference is small and likely not critical for clinical applications of the method.

The MBF values are slightly lower than that reported in some CMR studies and with other modalities such as PET. Model-independent and Fermi model methods also result in very similar perfusion estimates [28]. The relatively low perfusion values may be due in part from coil sensitivity- preliminary results with using proton density measurements to compensate for coil sensitivity variations tended to scale the blood pool region differently than the tissue, typically increasing MBF ~15%. As well, flow effects from arterial blood that has experienced fewer alpha pulses than the stationary tissue would tend to decrease MBF estimates due to increasing the AIF. This could be modeled with the equation used here if it could be determined how many fewer alpha pulses affected the blood pool. Adjustment for rate-pressure product [29] left mean MBF values virtually unchanged, with higher standard deviations: 0.67 ± 0.32 for the dual-bolus method, and 0.75 ± 0.33 for the multi-SRT method. The rate-pressure product adjusted MBFs did have a closer relationship: *MBF_multi-SRT _*= 0.97*MBF_dualbolus _*+ 0.1, r = 0.95.

Another source of error with the multi-SRT method are T_2_* effects during the first pass of contrast agent through the left ventricular blood pool [30]. In our study, T_2_* effects were assumed to be negligible for the moderate doses used. More study is needed to determine how well the method works with larger doses, and if T2* effects at the peak of the bolus can be accurately estimated [7].

## Conclusions

The multi-SRT estimation method using an undersampled radial k-space perfusion sequence accurately quantifies myocardial perfusion in the presence of 30-70% peak signal decrease due to saturation of the AIF. Unlike the dual-bolus imaging method, the multi-SRT method requires only a single contrast agent injection, which can greatly simplify myocardial perfusion studies, especially during stress imaging.

## Competing interests

The authors declare that they have no competing interests.

## Authors' contributions

TK performed image acquisition, image reconstruction, developed portions of the analysis, and created the initial draft of the manuscript. NAP participated in image acquisition, reconstruction, and analysis. LC performed key pulse sequence modifications and reconstruction design. EVRD conceived of the study, was involved with all aspects and edited the manuscript. All authors read and approved the final manuscript.
